# The Impact of Antiepileptic Drugs on Thyroid Function in Children with Epilepsy: New Versus Old

**Published:** 2020

**Authors:** Hatem Hamed ELSHORBAGY, Naglaa Fathy BARSEEM, Hany Abdelaziz SULIMAN, Eman TALAAT, Ashraf Hamed AlSHOKARY, Waleed Elsayed ABDELGHANI, Sameh Elsayed ABDULSAMEA, Yehia Hamed Abdel MAKSOUD, Sanaa Mohammed AZAB, Akram Elshafey ELSADEK, Dalia Mohamed NOUR EL DIN

**Affiliations:** 1 Pediatric department, Menofia University, Minufya, Egypt; 2 Pediatric department, Benha University, Minufya, Egypt; 3 Ain shams University, Pediatric department, Cairo, Egypt; 4 Department of Pediatric, Faculty of Medicine, Al-azhar University, Cairo, Egypt; 5 Clinical pathology-department, Benha University, Minufya, Egypt

**Keywords:** Antiepileptic drugs, Thyroid function, Subclinical hypothyroidism, Children

## Abstract

**Objectives:**

To investigate the effects of traditional antiepileptic drugs (AEDs) versus newer AEDs on the thyroid hormone profile of children with epilepsy.

**Materials & Methods:**

A total of 80 children with epilepsy were included in this study and were divided into two groups. Group 1 included 40 children with epilepsy on traditional AEDs, and group 2 included 40 children with epilepsy on newer AEDs. Forty healthy children were also included as the control group (group 3). We analyzed the serum levels of free triiodothyronine (FT3), free thyroxine (FT4), and thyroid stimulating hormone (TSH).

**Results:**

In epileptic children treated with traditional drugs, there was a significant reduction in the serum level of FT4 and a significant increase in TSH concentration, compared to the control group (*P*<0.001). Conversely, epileptic children treated with newer AEDs showed no significant changes in the serum concentrations of FT3, FT4, and TSH, compared to the control group.

**Conclusion:**

Traditional AEDs have more significant effects on thyroid hormone profile, compared to newer AEDs.

## Introduction

Epilepsy is a common neurological disorder, characterized by a disturbance in the electrical activity of the brain due to different factors. It includes many types of seizures with variable severity, seizure semiology, etiology, consequences, and management (1 ). It is a chronic disorder, which requires long-term therapy with antiepileptic drugs (AEDs). However, in some patients, seizures are resistant to AEDs despite appropriate treatment, and lifelong therapy is usually indicated (2 ).

Prolonged use of AEDs is associated with the increased risk of adverse events, such as drug interactions, cognitive dysfunction, idiosyncrasy, behavioral changes, and metabolic or endocrinal disturbances (2,3), particularly thyroid gland dysfunction (4). Thyroid hormones play an important role in the regulation of metabolism in many tissues. Therefore, disturbance of thyroid hormones has major implications for the growth and development of children (5).

The impact of AEDs on thyroid function is well-known in adults (6). However, disturbance of thyroid function in children receiving AEDs is a matter of controversy, and there is limited information in this context (4,7). Among drug therapies for epileptic children, most epileptologists preferred traditional AEDs, such as valproate, carbamazepine, and phenobarbital in the past. However, recently, major attention has been paid to newer drugs, such as levetiracetam, topiramate, oxcarbazepine, and lamotrigine. Newer AEDs are used as monotherapy, as well as add-on therapy for children with epilepsy (1) Previous studies have reported the effects of traditional AEDs on thyroid hormones in children. However, there is limited evidence regarding the effects of newer AEDs (8).

Several studies reported no change in the level of thyroid hormones among children with epilepsy receiving AEDs (9,10), while others indicated significant alterations in the level of thyroid hormones (11,12). Administration of AEDs can result in subclinical hypothyroidism, which is defined as the elevation of thyroid stimulating hormone (TSH) level above the upper limit of the age-specific reference range, while the serum level of free thyroxine (FT4) is within its reference range. 

Subclinical hypothyroidism appears to be a benign condition with a low risk of conversion into overt hypothyroidism. This condition has no adverse effects on neuropsychological functions (13-15) .Changes in thyroid function can be attributed to the administration of AEDs, especially traditional AEDs. Therefore, in this study, we aimed to investigate the effects of traditional AEDs versus newer AEDs on the thyroid hormone profile of children with epilepsy.

## Materials & Methods

This cross-sectional study was conducted on 80 children with epilepsy, including 48 males and 32 females. The participants’ age ranged from three months to 15 years. All patients were recruited from the pediatric neurology outpatient clinic of Al-Hada and Taif military hospitals in Saudi Arabia and had received AED therapy for more than six months with good compliance. The patients were divided into two groups: 40 children with epilepsy on traditional AEDs including valproate, carbamazepine, and phenobarbital (group 1); and 40 children with epilepsy on newer AEDs, including levetiracetam, oxcarbazepine, and topiramate (group 2). In addition, 40 age- and sex-matched healthy children were recruited as the control group (group 3). 

This study was conducted during February 2016 and August 2016 after obtaining informed consents from the participants. Ethical approval was also obtained for the study. The study protocol conformed to the ethical guidelines of 1964 Declaration of Helsinki and its later amendments. The exclusion criteria were as follows: 1) presence of an underlying etiology or drug-resistant seizures; 2) poor compliance; 3) polytherapy with more than one AED; 4) body mass index above the 95^th^ percentile for age and gender; 5) thyroxine replacement therapy and use of anti-thyroid drugs; 6) endocrinal, metabolic, or chronic medical disorders; and 7) symptoms suggestive of thyroid gland disorder.

Detailed history-taking, including demographic data, seizure type, epileptic syndrome, seizure etiology, onset age of epilepsy, selected AEDs, dosage of antiepileptic drugs, electroencephalographic (EEG) changes, and duration of AED therapy, was performed. Also, careful examination of thyroid glands for the presence of goiter was carried out.

**Analysis of thyroid function**

A 5-mL venous blood sample was taken from each participant while taking aseptic precautions. Blood samples were collected between 8 a.m. and 10 a.m. after overnight fasting. The samples were then centrifuged to separate the serum. The serum was analyzed using a chemiluminescence autoanalyzer for proper assay of thyroid hormone profile, including free triiodothyronine (FT3), free thyroxine (FT4), and TSH concentrations. Commercial enzymatic methods were also used for hormonal analyses (Immulite, Siemens) (16).

The thyroid hormone profile was interpreted according to age-specific reference ranges (−2, −1, 0, 1, and 2 SDS) for thyroid hormones in children(17 ).The serum level of valproic acid was measured via chemiluminescent immunoassay in an Immulite 2000 system (Siemens Medical Solutions, USA). The serum level of carbamazepine was also measured using florescence polarization immunoassay in an INTEGRA 400 system (Roche Diagnostics, USA). Finally, the serum level of phenobarbital was measured using an enzyme immunoassay in an Emit^®^ 2000 system (Siemens Healthcare Diagnostics, USA) ( 18).

**Statistical analysis**

Data and variables were analyzed using SPSS version 10.0 (Chicago, IL, USA). Values are expressed as mean±standard deviation (SD). One-way analysis of variance (ANOVA), followed by post hoc Dunnett's test, was used to evaluate significant differences between the groups. Qualitative data are also presented as number and percentage. To evaluate significant differences between the groups, Chi-square test was performed. *P*-value less than 0.05 was considered statistically significant.

## Results

Eighty epileptic children were enrolled in this study, including 48 males and 32 females. The participants’ age ranged from three months to 15 years. Group 1 included 40 epileptic children on traditional AEDs, including valproate, carbamazepine, and phenobarbital. The age of patients in this group ranged from three months to 12 years, with the mean age of 6.08±3.64 years. Group 2 included 40 epileptic children on newer AEDs, including levetiracetam, topiramate, and oxcarbazepine. The patients’ age in this group ranged from eight months to 15 years, with the mean age of 7.12±4.03 years. Also, group 3, which served as the control group, consisted of 40 age- and sex-matched healthy children. The mean age of the participants in this group was 6.58±3.56 years. 

Valproate was the most commonly used traditional AED, whereas levetiracetam was the most common AED among newer agents. Duration of drug therapy was 1.65±0.84 years in group 1 and 2.01±0.92 years in group 2. Among 40 epileptic children in group 1, 19 (47.5%) received valproate, 14 (35%) received carbamazepine, and 7 (17.5%) received phenobarbital. On the other hand, among 40 epileptic children in group 2, 21 (52.5%) received levetiracetam, 15 (37.5%) received topiramate, and 4 (10%) received oxcarbazepine. All AEDs were administered at appropriate doses with strict compliance.

**Figure (1):**
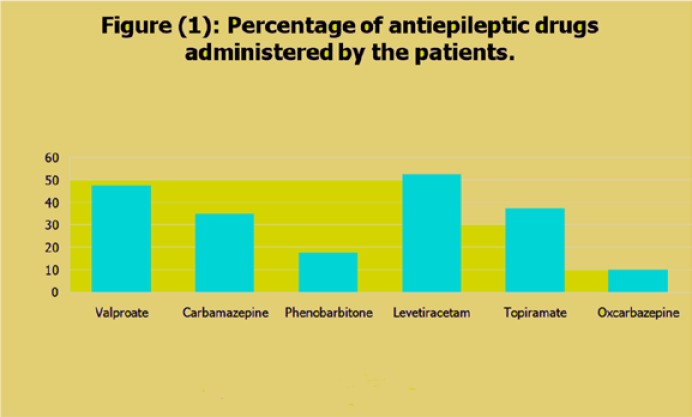
Percentage of antiepileptic drugs administered by the patients

EEG changes mostly included focal activity in form of sharp or spike waves and generalized spike-and-wave activity in both groups, while the background activity was normal. Serum concentrations of AEDs were within therapeutic ranges in all patients. Administration of different AEDs in patients is shown in Figure 1. 

With regard to the serum levels of FT3, FT4, and TSH, epileptic children treated with traditional drugs (group 1) showed a significant decrease in FT4 (0.76±0.13) and a significant increase in TSH concentration (3.61±1.36), compared to the control group (group 3) (FT4: 0.98±0.07; TSH: 2.15±0.81) (*P*<0.001). However, epileptic children treated with newer AEDs showed no significant changes in the serum concentrations of FT3, FT4, and TSH, compared to the control group (*P*=0.35, 0.24, and 0.61, respectively). Also, the results showed a significant difference between group 1 and group 2 in terms of serum concentrations of FT4 and TSH (*P*<0.01) (Table 1). Eight (20%) patients from group 1 (FT4: 0.96±0.08; TSH: 3.54±1.44) and two (5%) patients from group 2 (FT4: 0.94±0.08; TSH: 3.46±1.41) were diagnosed with subclinical hypothyroidism. Nevertheless, none of the controls showed evidence of subclinical hypothyroidism.

With regard to the prevalence of subclinical hypothyroidism in the studied groups, there was a significant difference between epileptic children treated with traditional drugs (group 1) and the control group (group 3) (*P*<0.01). However, the prevalence of subclinical hypothyroidism was not significantly different between epileptic children treated with newer drugs (group 2) and the control group (group 3) (*P*=0.19). Meanwhile, in epileptic children treated with traditional drugs (group 1), a significantly higher prevalence of subclinical hypothyroidism was reported, compared to epileptic children treated with newer drugs (group 2) (*P*<0.05) (Table 2).

**Table (1):** Serum level of thyroid hormone among studied groups

**Serum level of thyroid hormone**	**Epileptic children treated with traditional AEDs** **(Group1)**	**Epileptic children treated with newer AEDs** **(Group 2)**	**Controls** **(Group 3)**
fT3(pg/ml) *P*	2.30±0.65 0.38	2.54±0.28 0.74	2.43±0.26
fT4 (ng/dl) *P*	0.79±0.17 (*P* < 0.001)*.	0.96±0.08 0.58	1.02±0.24
TSH(mIU/ml) *P*	3.86±1.54 (*P* < 0.01)*.	2.16±0.87 0.83	2.23±0.26

Values are expressed as mean±SD for all variables. *: test the significance compared to control group(Group 3).The comparison was done by one-way ANOVA analysis followed by post hoc Dunnett's test. Group 1and Group 2 were compared to Group 3.fT3: free triiodothyronine, fT4: free thyroxine, TSH: thyroid stimulating hormone.

**Table (2):** Prevalence of subclinical hypothyroidism among studied groups

	**Epileptic children treated with traditional AEDs** **(Group1)**	**Epileptic children treated with newer AEDs** **(Group 2)**	**Controls** **(Group 3)**	***P***
Subclinical hypothyroidism	8 (20) (*P* < 0.01)*.	2(5) (*P* =0.19)*.	0(0)	(*P*<0.05

Values are expressed as number and percent. *: test the significance compared to control (Group 3). The comparison was done by chi^2^ test .*P*: test the significance between Group 1and Group 2.

## Discussion

While previous studies assessing thyroid function in epileptic children suggest that levels of thyroid hormones are not directly related to epilepsy, some changes in thyroid function may be attributed to the administered AEDs (19). It is well-established that thyroid hormones play an important role in different physiological processes. Hypothyroidism may progress into a metabolic syndrome with the involvement of many systems (20,21). Therefore, level of thyroid hormone needs to be measured and investigated thoroughly for epileptic children under long-term therapy with AEDs. 

Thyroid dysfunction has been associated with the administration of different AEDs. In our study, we found a significant decrease in the serum level of fT4 and an increase in the serum level of TSH (*P*<0.001) in epileptic children receiving traditional AEDs, compared to the control group. However, the serum level of fT3 was not influenced by the administration of AEDs (*P*=0.38). T3 is derived from the transformation of T4 in peripheral tissues. Serum levels of T3 and FT3 remain normal even in severe cases of hypothyroidism and are less sensitive in the diagnosis of hypothyroidism (22). Therefore, there was no significant change in the serum level of FT3 in our study. Our results are consistent with a study by Yilmaz et al, which showed a reduction in the serum level of FT4 and an increase in the serum level of TSH with valproate, carbamazepine, and phenobarbital administration, but not levetiracetam (8 ).Yehia et al. found similar results in their study (23).

In our study, we reported subclinical hypothyroidism in 20% of epileptic children treated with traditional drugs and 5% of epileptic children treated with newer drugs. However, none of the participants in the control group showed evidence of subclinical hypothyroidism. We found that none of our patients developed overt symptoms of hypothyroidism, and all patients were clinically euthyroid. According to previous studies, subclinical hypothyroidism may develop in epileptic patients during treatment with AEDs (24, 25). However, no symptoms or signs of hypothyroidism were reported.^24^ These changes did not affect the development of puberty among children. In other words, the prevalence of clinically manifest thyroid disorders is rare. However, these results may indicate the increased risk of hypothyroidism among epileptic patients treated with AEDs (27). 

Our findings showed a significantly higher prevalence of subclinical hypothyroidism in epileptic children treated with traditional drugs (group 1), compared to group 2 and group 3 (*P*<0.05 and *P*<0.01, respectively). In this regard, Yilmaz et al. and Sahu et al. reported a prevalence rate of 25% in epileptic children on valproate therapy (8,12). Subclinical hypothyroidism was detected in healthy control children with a prevalence of 0-7.7%. Generally, the association between epilepsy and altered thyroid function is not fully understood. Subclinical hypothyroidism has been reported in epileptic children before the onset of treatment; this may suggest that epilepsy plays a role in thyroid dysfunction (11, 12). 

Valproate, carbamazepine, and phenobarbital were the most commonly used traditional AEDs in our study. Previous studies showed a significant decrease in the serum level of FT4 and an increase in the serum level of TSH in patients treated with VPA; these changes were persistent throughout the study (8,11,28,29,30). In another study on adolescent girls with epilepsy, the group receiving valproate showed higher serum levels of TSH and lower serum levels of FT4, compared to the untreated group, although the values were still within the normal range (9). Other studies found that TSH level increased in patients using valproate, while FT4 level remained unchanged (12,28,31,32). On the other hand, several studies found that both FT4 and TSH concentrations were unaffected in patients treated with valproate (10,32). 

Similarly, previous studies indicated a significant decrease in the serum level of FT4 and an increase in the serum level of TSH in patients treated with carbamazepine (8,31). Other studies demonstrated that the serum level of FT4 significantly reduced in patients receiving carbamazepine, whereas the serum level of TSH remained unchanged in children (5,28,31,33). In concordance with our results, Yilmaz et al. reported similar effects of phenobarbital on thyroid hormones (8). Epileptic children on phenobarbital were found to have significantly reduced serum levels of FT4, despite unchanged serum TSH, compared to the controls, as reported in other studies (8,34 ).Conversely, another study indicated no significant differences in the serum levels of FT4 and TSH between patients receiving phenobarbital and the control group (35 ).

In line with our study, Adhimoolam and Arulmozhi reported similar findings by evaluating adult epileptic patients, who were administered traditional AEDs, including valproate, carbamazepine, and phenytoin (36).The controversial results regarding the effect of traditional AEDs on thyroid hormones in different studies can be partly attributed to differences in the study design and methods. The failure rate of the first AED prescription for patients with newly diagnosed epilepsy remains relatively high. This can be explained by the poor effectiveness and/or high frequency of side effects, which prompted further search for newer drugs. Generally, newer AEDs are better tolerated by patients, have few drug interactions, needless serum drug monitoring, and may have potential neuroprotective effects (36, 38). 

Introduction of new AEDs provides more options for the treatment of children with epilepsy. In an adult clinical trial, new AEDs, such as lamotrigine, oxcarbazepine, vigabatrin, levetiracetam, topiramate, zonisamide, felbamate, rufinamide, gabapentin, and perampanel, were approved as monotherapy and adjunctive therapy. Selection of a new AED for children with epilepsy depends on multiple factors, including age, etiology, cognitive development, and epileptic syndrome (39).Among our patients, levetiracetam, topiramate, and oxcarbazepine were commonly used. We observed no significant changes in the serum levels of FT3, FT4, and TSH (*P*=0.74, 0.58, and 0.83, respectively) by administration of these drugs in epileptic children, compared to the controls. Yilmaz et al. and Leskiewicz et al. reported similar effects of these newer drugs on thyroid hormones (8, 40). 

Evidence suggests that levetiracetam has fewer side effects than older AEDs. However, there is little evidence regarding the effects of levetiracetam on thyroid function. In a recent study, thyroid function was found to be unaffected in children receiving levetiracetam (35). Moreover, oxcarbazepine is structurally related to carbamazepine, but does not induce the same level of enzymatic activity. In adults, replacement of carbamazepine by oxcarbazepine can reverse the effects of carbamazepine on thyroid hormones (30). Previous studies found that the serum levels of fT4 were significantly reduced in epileptic children receiving oxcarbazepine, whereas the serum level of TSH remained unchanged (8, 28, 31, 33).

So far, no evidence has been reported regarding the effect of topiramate on thyroid hormones in epileptic children. Meanwhile, Adhimoolam and Arulmozhi observed no significant changes in the thyroid hormone profile of adult epileptic patients, who were administered newer AEDs, including topiramate, levetiracetam, and clobazam (36). Generally, the mechanism of association between AEDs and thyroid hormones is still unclear. It was postulated that alterations in the serum levels of thyroid hormones are caused by AEDs through different mechanisms. 

Most circulating thyroid hormones are bound to plasma proteins, and FT3 and FT4 do not appear to interfere with thyroxin-binding globulin. In addition, AEDs can cause impairment in thyroid-hormone homeostasis through changing their biosynthesis, secretion, metabolism, transport, and/or excretion (5). The most likely mechanism is attributed to the induction of hepatic enzyme, CYP450, by traditional AEDs (carbamazepine, phenobarbitone, and phenytoin), which is associated with the improved metabolism of thyroid hormones, resulting in the reduction of serum concentrations (31 ). Uridine 5'-diphospho-glucuronosyltransferase (UGT) is responsible for glucuronidation and plays a definite role in the metabolism of thyroid hormones. Some studies reported high levels of UGT after exposure to AEDs (41) Another mechanism might be related to interference in the hypothalamic-pituitary-thyroid axis, responsible for the regulation of thyroid hormone production (10). 

Carbamazepine can cause disturbance in thyroid hormones through inhibition of iodine uptake by the thyroid gland (42).We did not include a mechanistic approach in our study. However, based on our results, evaluation of thyroid hormone profile is recommended for epileptic children on long-term traditional AEDs, compared to newer drugs; therefore, complications due to thyroid dysfunction can be prevented. 

The main limitation of this study is the small sample size. Therefore, larger prospective studies are needed on patients to support our findings.

**In conclusion,** traditional AEDs have significant effects on the thyroid hormone profile of epileptic children on long-term therapy, compared to newer AEDs. Although these effects were not accompanied by clinical hypothyroidism, screening of thyroid hormone profile is recommended. Further prospective studies are recommended on a larger sample of patients to confirm our results.
